# Orthodontist and periodontist’s knowledge, attitudes and aspects of clinical practice, regarding fixed lower orthodontic retainers

**DOI:** 10.1590/2177-6709.26.4.e2119276.oar

**Published:** 2021-08-27

**Authors:** Ruth Suzanne Maximo da COSTA, Silvia Amélia Scudeler VEDOVELLO, Vivian Fernandes FURLETTI-GÓES, William CUSTODIO, Giovana Cherubini VENEZIAN

**Affiliations:** 1 Centro Universitário da Fundação Hermínio Ometto, Faculdade de Odontologia, Departamento de Ortodontia (Araras/SP, Brasil).

**Keywords:** Orthodontic retainers, Orthodontics, Periodontics, Dental biofilm, Knowledge

## Abstract

**Objective:**

This study aimed to assess the knowledge, attitudes, and aspects of the clinical practice of orthodontists and periodontists, regarding lower fixed orthodontic retainers.

**Methods:**

The orthodontists (n=502) and periodontists (n=269) who participated in this cross-sectional observational study received, via e-mail, questions related to the type of lower fixed retainer, dental biofilm accumulation, oral hygiene, and potential periodontal changes. The data were subjected to chi-square and Fisher’s exact tests, at 5% significance level.

**Results:**

Both orthodontists (72.3%) and periodontists (58.7%) reported that hygienic retainers accumulate more dental biofilm (*p*< 0.05), and 64.1% of orthodontists and 58.7% of periodontists considered that modified retainers may lead to periodontal changes (*p*< 0.05). There was no significant difference between the dental specialties, regarding the type of lower fixed retainer considered the easiest for the patient to perform hygiene (*p*> 0.05), whereas 48.6% of professionals chose the modified type.

**Conclusion:**

The modified retainer accumulates a greater amount of dental biofilm and, in the perception of orthodontists and periodontists, it may cause periodontal changes.

## RESUMO

**Objetivo:**

O objetivo do presente estudo foi avaliar o conhecimento, as atitudes e os aspectos da prática clínica de ortodontistas e periodontistas, com relação às contenções ortodônticas fixas inferiores.

**Métodos:**

Os ortodontistas (n = 502) e periodontistas (n = 269) que participaram desse estudo transversal observacional receberam, por correio eletrônico, perguntas relacionadas ao tipo de contenção fixa inferior e ao acúmulo de biofilme dentário, higiene bucal e possíveis alterações no periodonto. Os dados foram submetidos aos testes Qui-Quadrado e Exato de Fisher, com nível de significância de 5%.

**Resultados:**

Tanto os ortodontistas (72,3%) quanto os periodontistas (58,7%) relataram que a contenção higiênica acumula mais biofilme dental (*p*< 0,05), e 64,1% dos ortodontistas e 58,7% dos periodontistas consideram que a contenção modificada pode levar a alterações periodontais (*p*< 0,05). Não houve diferença significativa entre os especialistas a respeito do tipo de contenção fixa inferior considerado de mais fácil higienização pelo paciente (*p*> 0,05), sendo que 48,6% dos profissionais escolheram a do tipo modificada.

**Conclusão:**

Na percepção dos ortodontistas e periodontistas, a contenção modificada acumula maior quantidade de biofilme dentário, podendo causar alterações periodontais.

## INTRODUCTION

The use of retainers is desired at the end of orthodontic treatment, to prevent relapse of dental movements.^1^ ^-^ ^5^ Orthodontists are more likely to indicate fixed retainers adapted to the lower arch, because of tooth instability in the region, which requires longer stabilization periods.^1^ ^,^ ^2^ ^,^ ^6^ ^-^ ^10^ Fixed retainers are more aesthetic, do not depend on patient cooperation,^6^ ^,^ ^8^ ^,^ ^11^ ^,^ ^12^ and may be individualized for the diagnosis and treatment performed.^2^ ^,^ ^13^ ^,^ ^14^ In this context, the 3x3 fixed bar produced with straight wire bonded to the contralateral canines,^1^ ^,^ ^12^ ^,^ ^14^ the twisted wire bonded to all lower anterior teeth,^1^ ^,^ ^9^ ^,^ ^12^ ^,^ ^15^ ^,^ ^16^ ^,^ ^17^ and the modified fixed retainer^1^ ^,^ ^12^ ^,^ ^14^ ^,^ ^16^ are the mostly used.

Although acknowledging the benefits of using retainers in orthodontics, studies affirm that dental biofilm accumulation increases with the use of all types of fixed retainers, requiring constant periodontal health assessments to prevent potential periodontal changes.^10^ ^,^ ^13^ ^,^ ^17^ ^,^ ^20^

Clinical studies analyzing periodontal parameters after using different types of lower anterior fixed orthodontic retainers have highlighted the difference in biofilm retention, and the risk of developing periodontal changes in these patients.^14^ ^,^ ^16^ ^-^ ^18^ However, the cost-benefit ratio of the clinical use of different types of orthodontic retainers has not been defined yet, and there are no studies comparing the advantages and disadvantages of each type of retainer.

Seeking to highlight the existence of cost-benefit ratio differences among the lower fixed retainers mostly used today, and to contribute to orthodontist selection of the retainer type, this study aimed to assess the knowledge, attitudes, and aspects of the clinical practice of orthodontists and periodontists, regarding lower fixed orthodontic retainers.

## MATERIAL AND METHODS

The Human Research Ethics Committee of *Centro Universitário da Fundação Hermínio Ometto* approved this study (protocol #71249317.0.0000.5385).

This was a national cross-sectional observational study performed with orthodontists and periodontists. A structured questionnaire was created to assess the knowledge, attitudes, and clinical practices of dentists. Initially, the questionnaire was sent via e-mail to 2,553 dentists specialized in orthodontics (n = 1,565) or periodontics (n = 988). The collection ended 60 days after the initial e-mail was sent, and the data were stored in the Google Forms digital platform.

A total of 850 dentists eligible for the study filled out and returned the questionnaires, which had a final response rate of 33.3%, including 548 orthodontists and 312 periodontists. Seventy-nine questionnaires were excluded due to incomplete information. Thus, the final sample included 771 professionals: 502 orthodontists and 269 periodontists. The sample size provided a test power above 80% at 5% significance level, in all analyses of association of professional specialty with knowledge and performance on lower fixed orthodontic retainers. The analyses were performed in the R Core Team software (R Foundation for Statistical Computing, Vienna, Austria).

**Figure 1:**
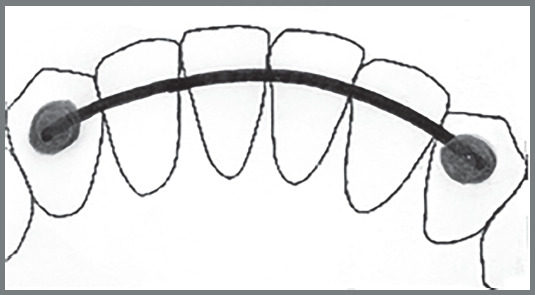
3x3 bar with straight wire.

**Figure 2:**
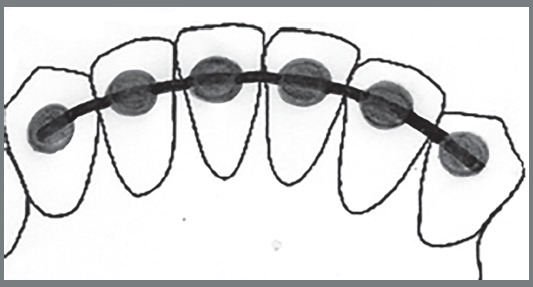
3x3 bar with twisted wire.

**Figure 3:**
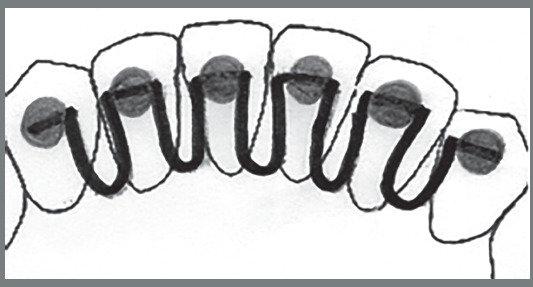
Modified 3x3 bar.

The instrument consisted of a drawing, a brief description of the lower fixed retainers - 3x3 bar with straight wire (Fig 1), 3x3 bar with twisted wire (Fig 2), and modified 3x3 bar (Fig 3) -, and nine questions related to knowledge, attitudes, and clinical practice on using retainers (Table 1).

**Table 1:** Questionnaire.

Lower fixed orthodontic retainer questionnaire
1. What type of lower fixed retainer do your patients mostly use?
2. What retainer do you consider the easiest for the patient to perform hygiene?
3. What retainer do you believe accumulates more dental biofilm?
4. Do you believe that using lower fixed retainer may cause periodontal change?
5. What type of retainer do you believe might cause periodontal change?
6. Do you believe that the number of teeth fixed (bonded) to the retainer may lead to periodontal changes?
7. What means of bonding do you believe may cause periodontal changes?
8. How long do you consider ideal for performing prophylaxis and scaling after installing the retainer?
9. How long do you think the patient should use the retainer?

## STATISTICAL ANALYSIS

Absolute and relative frequency distribution tables were produced. Chi-square and Fisher’s exact tests analyzed the associations between the answers and professional specialties, at 5% significance level. All analyses were performed in the R Core Team software (R Foundation for Statistical Computing, Vienna, Austria).

## RESULTS

The final sample included 771 specialists, including 502 orthodontists and 269 periodontists. Table 2 presents the association of knowledge, attitudes, and clinical practice of orthodontists and periodontists, regarding the use of lower fixed orthodontic retainers. It was verified that the mostly used retainer, for both specialties, was the straight wire type (*p*< 0.05). The retainer that dentists believe accumulate the greatest amount of dental biofilm is the modified one, considered by 72.3% of orthodontists and 58.7% of periodontists (*p*< 0.05). However, 48.4% of orthodontists and 49.1% of periodontists considered the modified retainer the easiest design for the patient to perform hygiene (*p*> 0.05).

**Table 2:** Association of knowledge, attitudes, and clinical practice of orthodontists and periodontists regarding the use of lower fixed orthodontic retainers.

Category		Specialty			p-value
		Total	Orthodontics	Periodontics	
		n (%)	n (%)	n (%)	
Mostly used retainer	Straight wire	369 (47.9)	248 (49.4)	121 (45.0)	0.0187
	Twisted wire	199 (25.8)	138 (27.5)	61 (22.7)	
	Modified	203 (67.6)	116 (23.1)	87 (32.3)	
Perception of dental biofilm accumulation	Straight wire	65 (8.4)	30 (6.0)	35 (13.0)	0.0001
	Twisted wire	185 (24.0)	109 (21.7)	76 (28.3)	
	Modified	521 (67.6)	363 (72.3)	158 (58.7)	
Easiest retainer for the patient to perform hygiene	Straight wire	488 (39.6)	197 (39.2)	108 (58.7)	0.8114
	Twisted wire	91 (11.8)	62 (12.4)	29 (10.8)	
	Modified	375 (48.6)	243 (48.4)	132 (49.1)	
Prophylaxis and scaling after retainer installation	Up to 3 months	391 (50.7)	184 (36.7)	207 (77.0)	<0.0001
	3 to 6 months	361 (46.8)	300 (59.8)	61 (22.7)	
	1 year	19 (2.5)	18 (3.6)	1 (0.4)	
Retainer causes periodontal damage	No	230 (29.8)	180 (35.9)	50 (18.6)	<0.0001
	Yes	541 (70.2)	322 (64.1)	219 (81.4)	
Type of retainer that causes periodontal damage	Straight wire	69 (8.9)	42 (8.4)	27 (10.0)	0.0077
	Twisted wire	171 (22.2)	97 (19.3)	74 (27.5)	
	Modified	480 (62.3)	322 (64.1)	158 (58.7)	
	None	51 (6.6)	41 (8.2)	10 (3.7)	
Number of teeth bonded may cause periodontal damage	No	292 (37.9)	223 (44.4)	69 (25.7)	<0.0001
	Yes	479 (62.1)	279 (55.6)	200 (74.3)	
Type of retainer	Only on canines	192 (24.9)	116 (23.1)	76 (28.3)	<0.0001
	All anterior teeth	500 (64.9)	316 (62.9)	184 (68.4)	
	Regardless of bonded teeth	79 (10.2)	70 (13.9)	9 (3.3)	
Time of retainer use	Up to 6 months	45 (5.9)	9 (1.8)	36 (13.4)	<0.0001
	6 months to 1 year	99 (12.8)	42 (8.4)	57 (21.2)	
	Depends on the professional	59 (7.7)	20 (4.0)	39 (14.5)	
	Does not recommend removal	568 (73.7)	431 (85.9)	137 (50.9)	

Still, according to Table 2, there was a difference in professional approach regarding the time to perform prophylaxis and scaling after installing the retainer: Most periodontists (77.0%) indicate up to three months, while orthodontists (59.8%) prefer three to six months (*p*< 0.05). Although most dentists believe that using lower fixed retainers may cause periodontal damages, periodontists (81.4%) reported it more than orthodontists (64.1%). Moreover, 64.1% of orthodontists and 58.7% of periodontists considered that the modified retainer causes more damages to periodontal health (*p*< 0.05). Differences were also verified when considering the number of teeth bonded to the retainer, regarding periodontal damage (*p*> 0.05): 62.9% of orthodontists and 68.4% of periodontists (*p*< 0.05) believe that bonding to every tooth may cause more periodontal changes. It was also noted that most orthodontists (85.9%) and half of the periodontists (50.9%) affirmed they do not recommend removing orthodontic retainers (*p*< 0.05).

## DISCUSSION

Lower fixed orthodontic retainers provide stability to tooth positioning after the end of orthodontic treatment, alongside the action of periodontal readaptation forces.^5^ ^,^ ^19^ Therefore, it is essential to know the attitudes and the clinical practice of orthodontists and periodontists, because understanding potential differences may contribute to guide the clinical practice of both type of professionals. Thus, this study chose to include all orthodontists and periodontists, aiming at a more extensive population sample.

The findings of the present study showed that most orthodontists and periodontists consider that the modified retainer accumulates a greater amount of dental biofilm. According to the professionals, the accumulation may be related to wire curvature in the cervical third, and to the use of a greater amount of orthodontic wire, as reported in previous studies.^14^ ^,^ ^16^ The professionals also considered the modified retainer as the type that causes more periodontal damages, presenting higher difficulty to perform oral hygiene, especially because it is bonded to all dental elements, corroborating clinical studies that identified greater biofilm accumulation in this type of retainer.^17^ ^,^ ^18^ However, the literature has reported that, because such retainer has free interproximal areas, it is easier for the patient to perform oral hygiene, especially for using dental floss.^17^ ^,^ ^20^ ^,^ ^21^

Orthodontists and periodontists reported the 3x3 fixed retainer with straight wire as the mostly used type. This choice may be related to the ease of production and for considering this retainer to cause less periodontal damage, which may influence the preference of periodontists for it. The preference of orthodontists for this type of retainer had already been reported in previous studies.^1^ ^,^ ^12^

It is also worth noting that the use of orthodontic retainer, in the opinion of orthodontists (64.0%) and periodontists (82.0%), may cause periodontal damages. However, retainers are indicated because of the action of periodontal ligament fibers, which tend to move the tooth to its original position, before orthodontic treatment, and induce relapse after removing the orthodontic appliance.^22^ It was also verified that most orthodontists (84.5%) do not recommend removing lower fixed orthodontic retainers. Among periodontists, 49.8% do not recommend removing the retainer, and 21.7% recommend the removal after six months to one year, because of the potential periodontal damages. The concern with periodontal integrity related to retainers is based on scientific evidence showing that individuals who had never used orthodontic retainers presented a lower rate of clinical attachment loss and drilling depth in the interproximal surfaces, when compared to patients using lower fixed retainers.^23^

In order to prevent periodontal changes, most periodontists recommend performing prophylaxis and scaling up to three months after installing the retainer, but orthodontists believe that the time most indicated is between three and six months. Considering the potential for bacterial colonization in the dental biofilm, each patient should be assessed individually to determine the time to perform prophylaxis and scaling.

Finally, it is important to emphasize that the choice of retainer affects biofilm accumulation and the hygiene challenges of the patient, which may even lead to periodontal changes such as clinical attachment loss and increased drilling depth. There is no ideal type of retainer. The results of this study showed that professionals, both orthodontists and periodontists, are aware of the importance of the use of retainers and its limitations. It is also highlighted that professionals are in charge of assessing individually their cost-benefit, considering oral hygiene and the time of use for each patient, as well as determining the need for professional prophylaxis and scaling, which may vary among patients.

Considering that this study has only assessed the opinion of professionals on fixed orthodontic retainers, further studies are suggested to assess means of performing oral hygiene by patients using orthodontic retainers and the level of toothbrushing of such patients.

## CONCLUSION

Orthodontists and periodontists agree that the several types of retainers are different regarding biofilm accumulation, considering that the 3x3 bar with straight wire accumulates less biofilm, followed by the twisted wire retainer, which are easier for performing professional hygiene.
